# Physical activity and exercise alter cognitive abilities, and brain structure and activity in obese children

**DOI:** 10.3389/fnins.2022.1019129

**Published:** 2022-10-20

**Authors:** Xueyun Shao, Li Hai Tan, Longfei He

**Affiliations:** ^1^School of Sports, Shenzhen University, Shenzhen, China; ^2^Shenzhen Institute of Neuroscience, Shenzhen, China

**Keywords:** neuroscience, childhood obesity, physical activity, exercise, brain structure, neural activity, cognitive functioning

## Abstract

The prevalence of childhood obesity is increasing to such an extent that it has become a major global public health problem in the 21st century. Obesity alters children’s brain structure and activity and impairs their cognitive abilities. On the basis of these findings, it is necessary for educational and healthcare institutions to combat childhood obesity through preventive and therapeutic strategies. In general, exercise and physical activity are considered common but effective methods for improving physical, psychological, and brain health across the life span. Therefore, this review article mainly focuses on existing neuroimaging studies that have used magnetic resonance imaging (MRI), and functional magnetic resonance imaging (fMRI)to assess children’s brain anatomy and neural activity. We intended to explore the roles of physical activity and exercise in modulating the associations among childhood obesity, cognitive abilities, and the structure and activity of the brain.

## Introduction

According to the World Health Organization (WHO), an estimated 41 million children under the age of 5 years were overweight or obese in 2016 (NCD Risk Factor Collaboration (NCD-RisC), 2017). The prevalence of childhood obesity continues to increase in both developed and developing countries. In the United States, nearly one-fifth of children are obese, and in many countries, the rate of obesity has risen even higher in children than in adults (GBD 2015 Obesity Collaborators et al., 2017).

Obesity, the excessive accumulation of body fat or adiposity, is considered a complex and multifactorial health problem (Ng et al., 2014) that may lead to several comorbidities, including insulin resistance, hypertension, and type 2 diabetes (Ortega et al., 2016a). Obesity has induced a series of negative changes in brain structure and function that have been discovered using neuroimaging techniques; moreover, these obesity-induced alterations in the brain are correlated with poorer cognitive function (Sui and Pasco, 2020; Tanaka et al., 2020). Obesity is found to persist from childhood to adulthood (Simmonds et al., 2015); moreover, childhood obesity is closely associated with higher risk for cardiovascular disease, metabolic disease and cancer in adulthood (Llewellyn et al., 2016; Weihrauch-Blüher et al., 2019; Weihe et al., 2020; Horesh et al., 2021). A study using a rodent model observed that early exposure to obesity and insulin resistance may have long-term deleterious consequences in the brain, contributing to cognitive dysfunction during aging (Wang et al., 2015). The pathogenesis of obesity is defined as an imbalance between food intake and energy expenditure (Gómez-Apo et al., 2021). The interaction between the gut and the brain is essential for energy homeostasis. Some strains of bacteria and their metabolites target the brain directly and regulate central appetite and food reward signaling (Torres-Fuentes et al., 2017; van Son et al., 2021). Healthy body weight is coordinated by the strictly regulated balance between intestinal, extraintestinal and central homeostatic and hedonic mechanisms (Berthoud et al., 2017; Frank et al., 2021).

Given the high prevalence and negative consequences of childhood obesity, developing comprehensive therapeutic strategies for obese children is imperative (Brown et al., 2019). During childhood, the brain undergoes developmental changes in the gray and white matter structures, which may be followed by changes in cognition. In early childhood, which is defined as the period between term birth and 2 years old, the gray matter volume increases rapidly compared with the white matter volume (Gilmore et al., 2018). As the brain develops throughout childhood and adolescence, a pattern of gray matter contraction and white matter expansion appears (Bray et al., 2015). In this sensitive period of maturation, several health-related behaviors, such as physical activity (PA) and exercise have been found to be related to brain development and cognitive abilities (Gomes da Silva and Arida, 2015; Segalowitz, 2016; Bidzan-Bluma and Lipowska, 2018). PA, is defined as any movement produced by a human’s skeletal muscles that increases energy expenditure above the resting value. Exercise is a planned, structured, systematic and purposeful subcategory of PA (Dasso, 2019), and is considered a common but effective method for people to improve physical and mental health across the life span (Ruegsegger and Booth, 2018; Schuch and Vancampfort, 2021). Previous research has elucidated the effects of PA and exercise on brain and cognition in healthy normal-weight children (Kramer and Erickson, 2007; Hillman et al., 2008), but few studies have focused on overweight and obese children.

Magnetic resonance imaging (MRI) is a noninvasive technique that is used to obtain images of the structures of many organs, including the brain, and it provides comprehensive, multiparametric information on brain anatomy (Yousaf et al., 2018). Functional magnetic resonance imaging (fMRI), which is a widely applied method of neuroimaging in cognitive neuroscience, is of course based on MRI (Logothetis, 2008). fMRI shows task-induced changes in the deoxyhemoglobin concentration and thus can detect neural activity while participants perform cognitive tasks (Glover, 2011). In this review, we focused on neuroimaging studies using MRI to assess children’s brain structure and fMRI to assess children’s neural activity. We summarized the key findings of the neuroimaging studies in this literature review (see Table 1).

**TABLE 1:** Summary and key findings of the neuroimaging studies in this literature review.

References	Aim	Sample	Experimental design	Main results
Ou et al., 2015	Examine the differences in gray and white matter structure between obese and normal-weight children	12 obese and 12 normal-weight children (8–10 years old)	Group comparison of regional gray and white matter between two groups.	Obese children showed decreased gray matter volume in the MTG, SPL, PrCG, PCG, cerebellum, and thalamus. They also showed increased FA in white matter tracts in the left cerebral hemisphere.
Ronan et al., 2020	Examine whether differences in cortical thickness mediate the relationship between childhood obesity and executive function	3,923 healthy children (9–11 years old)	Mediation analysis: regional cortical thickness as a mediator, executive function as the dependent variable, and BMI as the independent variable.	Cortical thickness was a significant mediator of the relationship between BMI and executive function.
Gracia-Marco et al., 2020	Examine the associations of LMI with regional gray and white matter volume in overweight and obese children	100 overweight or obese children (10.0 ± 1.1 years old)	Multiple regression models were used to assess the relationships of LMI with gray and white matter volume.	LMI was positively related to the gray matter volume in the superior frontal and -orbital gyri and in the cerebellum. LMI was also positively associated with white matter volumes in frontal, parietal, and subcortical regions, as well as the cerebellum.
Migueles et al., 2020	Examine the associations between objectively measured PA, SED, and hippocampal gray matter volume in pediatric obesity	93 overweight or obese children (10.0 ± 1 years old)	Participants wore wrist-mounted accelerometers for 7 consecutive days, and PA and SED time were calculated from the accelerometer data.	MVPA time was positively associated with gray matter volume in the right HPC in children with obesity type I, while SED was negatively related to gray matter volume in the right HPC in overweight children.
Rodriguez-Ayllon et al., 2020a	Examine the associations of objectively measured and self-reported PA and SB with the white matter microstructure in overweight or obese children	103 overweight or obese children (10.02 ± 1.15 years old)	Objectively measured and self-reported PA and SB were assessed using accelerometers and the Youth Activity Profile-Spain questionnaire, respectively.	Objectively measured LPA, MVPA, and total PA were positively related to global FA. Self-reported total PA was positively related to global FA, while watching television, a self-reported SB, was negatively correlated with global FA.
Zavala-Crichton et al., 2020	Examine the associations of different self-reported SBs with gray matter volume in overweight or obese children	99 overweight or obese children (10.0 ± 1 years old)	SB was measured using the Youth Activity Profile Spain questionnaire.	Watching television was negatively linked with gray matter volume in frontal, parietal, and occipital regions, while playing more video games was associated with reductions in gray matter volume in temporal regions.
Mora-Gonzalez et al., 2019a	Examine the associations of PA and SED with executive function in overweight and obese children	96 overweight or obese children (10.0 ± 1 years old)	PA and SED were assessed using accelerometers. In addition, executive function was assessed through the Delis–Kaplan Executive Function System.	No significant association was observed between physical activity or; sedentary time and any domain of executive function in overweight and obese children.
Mora-Gonzalez et al., 2019b	Examine the association of PA with working memory and neuroelectric activity in overweight or obese children	79 overweight or obese children (10.0 ± 1.1 years old)	PA was assessed using accelerometers. Working memory was assessed using the delayed nonmatching-to-sample task. Neuroelectric activity was assessed using electroencephalogram.	Vigorous physical activity was correlated with a higher response accuracy of the working memory task and with a larger P3 amplitude in overweight and obese children.
Mora-Gonzalez et al., 2020	Examine the association of PA with inhibitory control and neuroelectric activity in overweight or obese children	84 overweight or obese children (8–11 years old)	PA was assessed using accelerometers. Inhibitory control was evaluated using a flanker task. P3 amplitude and latency were recoded using electroencephalogram.	Physical activity (moderate, vigorous, and moderate to-vigorous) was associated with inhibitory control and the underlying brain activity in overweight and obese children.
Tomporowski et al., 2008	Examine the effects of a bout of moderate aerobic exercise on overweight children’s executive functioning	69 overweight children (9.2 ± 1.2 years old)	Exercise group: a 23 min treadmill walk. Control group: remained sedentary and watched an age-appropriate video for 23 min. Cognitive task group: a visual switching task.	An acute bout of exercise had no impact on task switching performance in the exercise group.
Raine et al., 2020	Examine the effects of acute PA on inhibitory control task performance	116 normal weight children (9.88 ± 0.06 years old)	Exercise group: a 20 min treadmill walk. Resting control group: reading for 20 min. Cognitive task: a flanker task that required inhibitory control.	Children showed improved inhibitory control after an acute moderate aerobic exercise in the exercise group. These beneficial outcomes were attenuated in children with a higher BMI.
Davis et al., 2007	Examine the effects of aerobic exercise on overweight children’s cognitive functioning	94 overweight children (9.2 ± 0.84 years old)	Intervention schedule: 5 days per week for 15 weeks. Groups: low-dose (20 min/day) exercise group, high-dose (40 min/day) exercise group, and nonexercise control group. Cognitive performance measurement tool: Cognitive Assessment System.	The control group had a decreased posttest score relative to the high-dose exercise group; these group differences appeared only on the planning scale.
Davis et al., 2011	Examine whether aerobic exercise improves executive function and alters brain activation in overweight children	171 overweight children (9.3 ± 1.0 years old)	The exercise intervention program was the same as Davis et al. (2007). fMRI cognitive task: an antisaccade task. Academic achievement: the Woodcock–Johnson Tests of Achievement III.	Increased bilateral PFC activity and decreased bilateral PPC activity were found in the exercise group. There was no significant difference in mathematics achievement between the exercise group and the control group.
Krafft et al., 2014a	Examine the effect of an exercise intervention on brain activation during two cognitive control tasks in overweight children	43 overweight children (8–11 years old)	The intervention lasted 40 min per day for 8 months. The exercise group took part in aerobic activities. The attention control group participated in sedentary activities. The fMRI cognitive tasks comprised antisaccade and flanker tasks.	No group-by-time interactions were observed in adiposity in the participants. The exercise group showed increased activation overtime in brain regions contributing to flanker tasks, including the ACC and SFG, and decreased activation overtime in regions contributing to antisaccade tasks, including the PrCG and PPC.
Schaeffer et al., 2014	Examine whether exercise alters white matter integrity in overweight children	18 overweight children (8–11 years old)	The exercise intervention program was the same as in the study by Krafft et al. (2014a).	From baseline to posttest, the exercise group showed significantly decreased body fat compared with the control group. In the posttest, the exercise group had significantly higher FA values in bilateral UF and lower RD values in left UF than the sedentary control group, normalized to baseline.
Krafft et al., 2014b	Examine the association between participants’ attendance at an intervention program and frontoparietal white matter integrity in overweight children	18 overweight children (8–11 years old)	The exercise intervention program was the same as in the study by Krafft et al. (2014a).	In the exercise group, higher attendance was correlated with increased white matter integrity, that is, increased FA and decreased RD, in the bilateral SLF compared with the control group.
Krafft et al., 2014c	Examine the effect of an exercise intervention on resting-state synchrony in overweight children	22 overweight children (8–11 years old)	The exercise intervention program was the same as in the study by Krafft et al. (2014a).	The exercise group showed decreased synchrony overtime in the default mode network, cognitive control network and motor network, whereas synchrony was increased between the motor network and the frontal lobe.

MTG, middle temporal gyrus; SPL, superior parietal lobule; PrCG, precentral gyrus; PCG, postcentral gyrus; HPC, hippocampus; PFC, prefrontal cortex; PPC, posterior parietal cortex; ACC, anterior cingulate cortex; SFG, superior frontal gyrus; UF, uncinate fasciculus; SLF, superior longitudinal fasciculus; FA, fractional anisotropy; RD, radial diffusivity; BMI, body mass index; LMI, lean mass index; PA, physical activity; LPA, light physical activity; MVPA, moderate to vigorous physical activity; SED, sedentary time; SB, sedentary behavior; fMRI, functional magnetic resonance imaging.

The literature search was performed in PubMed, Google Scholar, and Web of Science with the key terms “childhood obesity,” “exercise,” “physical activity,” “brain structure,” “brain activation,” and/or “cognitive function.” Several abbreviations are used in this article; therefore, we have included an abbreviation list (see Table 2).

**TABLE 2:** Abbreviations.

Abbreviations	Full name
MRI	Magnetic resonance imaging
fMRI	Functional magnetic resonance imaging
WHO	World Health Organization
DTI	Diffusion tensor imaging
CDC	Centers for Disease Control and Prevention
BMI	Body mass index
FA	Fractional anisotropy
ABCD	Adolescent Brain Cognitive Development
VFR	Visceral fat ratio
LMI	Lean mass index
PA	Physical activity
SED	Sedentary time
LPA	Light physical activity
MVPA	Moderate to vigorous physical activity
SB	Sedentary behavior
YAP-S	Youth Activity Profile Spain
CAS	Cognitive Assessment System
DXA	Dual-energy X-ray absorptiometry
RD	Radial diffusivity
SLF	Superior longitudinal fasciculus
FGF21	Fibroblast growth factor 21
ADHD	Attention deficit and hyperactivity disorder

In this review, we first demonstrate obesity-related alterations in the brain and cognitive abilities during childhood. Next, we discuss the association of PA with brain structure and cognitive functioning in overweight and obese children. Following this, we examine the effects of exercise on cognition, brain structure and activation. Finally, current knowledge is summarized and possible future directions are discussed.

## Obesity-related alterations in the brain and cognitive abilities during childhood

**FIGURE 1:**
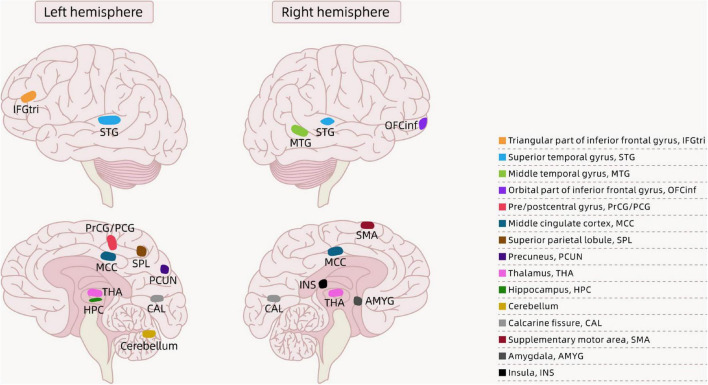
Brain regions showing the differences between obese children and normal-weight children in the literature.

In recent years, there have been growing concerns about childhood obesity, which can impact children’s health and even their quality of life. Obesity is associated with brain structure changes (i.e., gray matter volume, white matter integrity, and cortical thickness), as well as cognitive abnormalities in children. Ou et al. (2015) assessed the gray and white matter structures of 12 obese children and 12 healthy normal-weight children aged 8–10 years using T1-weighted three-dimensional MRI and diffusion tensor imaging (DTI) methods. The Centers for Disease Control and Prevention (CDC) defines the following classification based on age- and sex-adjusted body mass index (BMI) percentiles for children: underweight <5th percentile; healthy weight: 5th to <85th percentile; overweight: 85th to <95th percentile; and obese: 95th percentile or higher (Centers for Disease Control and Prevention, 2016). In a comparison between obese and normal-weight children, researchers found that obese participants had significant regional gray matter volume reductions in the right middle temporal gyrus, left superior parietal lobule, left pre- and postcentral gyri, left cerebellum, and bilateral thalamus. The results also showed that compared with their normal-weight peers, obese children showed increased regional white matter volume in a widespread array of brain regions, mainly consisting of the bilateral orbitofrontal, inferior/medial/superior temporal, and superior frontal white matter; the anterior and posterior limbs of the internal capsule; and the external capsule. In addition, the authors found increased fractional anisotropy (FA) values in multiple white matter tracts in the left cerebral hemisphere, and a positive association between BMI and FA in several white matter tracts. Furthermore, these findings, which showed reductions in gray matter in obese children compared with their healthy weight peers, are in agreement with the studies by Bauer et al. (2015) and Jiang et al. (2022). Bauer et al. (2015) found a reduced left hippocampal volume in obese children compared with normal-weight children. Jiang et al. (2022) found that obese children had greater reductions in gray matter volume in the prefrontal lobe, thalamus, right precentral gyrus, caudate, and parahippocampal gyrus/amygdala than normal-weight children. We created a figure that shows the regions of reduced gray matter volume in obese children according to the findings of Ou et al. (2015), Bauer et al. (2015), and Jiang et al. (2022) (see Figure 1).

In addition to changes in gray and white matter structure, obesity is associated with children’s cortical thickness. Ronan et al. (2020) investigated MR-derived measures of cortical thickness for 2,700 children aged 9–11-years from the Adolescent Brain Cognitive Development (ABCD) dataset. The ABCD dataset is derived from a study of over 10,000 children recruited from 21 sites in the US in 2017; this study focused on childhood brain development and employed structural MRI methods to image the structures of children’s brains. Researchers used the National Institutes of Health Toolbox Cognition Battery to evaluate each child’s composite score for executive function, which was corrected for age. The authors also conducted a mediation analysis to detect whether cortical thickness mediated the relationship between BMI and executive function. This study found a negative correlation between children’s BMI and age-corrected executive function. And increased BMI was linked to a thinner cortex in a wide range of brain regions, especially the prefrontal cortex, which is an area related to executive function. Moreover, a lower composite score on tests of executive function was also associated with a lower reduced mean global cortical thickness. Finally, cortical thickness was a significant mediator of the relationship between BMI and executive function. Laurent et al. (2020) also using the ABCD dataset and similar analysis methods, found substantially similar results. In contrast, Saute et al. (2018) showed contrary results regarding cortical thickness in obese adolescents aged 15–18 years. They found no association between BMI and cortical thickness in any brain region, whereas a higher visceral fat ratio (VFR), an indicator of intra-abdominal fat, was linked to greater cortical thickness.

Body mass index is an indicator of body composition that helps determine whether a person is obese, overweight, normal weight, or underweight. In recent years, several studies have focused on the lean mass index (LMI), which is a measurement focused on lean body tissue. Gracia-Marco et al. (2020) conducted a cross-sectional study as a part of the ActiveBrains project. The ActiveBrains project was a randomized controlled trial that aimed to examine the effects of an exercise program on brain, cognitive and academic performance and evaluated physical and mental health outcomes in 110 overweight and obese children aged 8–11 years in Granada, Spain. In this cross-sectional study, researchers collected baseline data on body composition and brain volume in 100 overweight or obese children. Structural brain images were captured from participants by using MRI. This study showed that LMI was positively correlated with regional gray matter volume in the superior frontal and -orbital gyri and in crus I of the cerebellum, yet BMI was not correlated with gray matter volume in any brain regions in overweight children. Additionally, higher BMI was linked to greater white matter volume in the gyrus rectus and cerebellar lobule VIII, and higher LMI was associated with greater white matter volume in a wide range of brain regions, including frontal regions, parietal regions, subcortical regions, and the cerebellum, in overweight or obese participants. These findings are in agreement with a study indicating that decreased lean mass was related to brain atrophy in early Alzheimer’s disease populations (Burns et al., 2010).

## Physical activity, brain structure, and cognitive functioning in overweight and obese children

Physical activity, a powerful marker of children’s physical health, is defined as any movement produced by human skeletal muscles that increases energy expenditure from the resting value. Sedentary behavior consists of any behavior performed when individuals are in a resting state, such as a seated or recumbent posture. Overweight or obese children perform less PA and engage in more sedentary behavior (SB) than normal-weight children (Wafa et al., 2016). Several observational studies have found relationships among PA, brain structure, and cognition.

In the study by Migueles et al. (2020), researchers collected data on children’s PA using accelerometers. Accelerometers are small wearable monitors that objectively measure PA (Ainsworth et al., 2015). Accelerometer technology facilitates the assessment of accelerations produced by movement and is regarded as an effective instrument to measure PA levels. Accelerometers objectively measure the frequency, duration and intensity of PA; however, they are expensive, require time, and are difficult to administer in a large population (Marasso et al., 2021). Participants wore an accelerometer on the nondominant wrist for 7 consecutive days and completed a sleep log where they recorded the time they went to bed and the time they rose every day; the sleep logs were used to calculate the children’s daily sleeping duration. Children’s daily sedentary time (SED), light physical activity (LPA), and moderate to vigorous physical activity (MVPA) were calculated using the data from accelerometers. Structural brain images were acquired with MRI. The authors observed a positive association between the objectively assessed MVPA time and gray matter volume in the right hippocampus of children with obesity type I. Specifically, replacing 20 min/day of SED with MVPA would be correlated with 100 mm^3^ more gray matter volume in the right hippocampus. Relative to LPA and MVPA, a longer time spent in objectively assessed SED was associated with lower gray matter volume in the right hippocampus in overweight children. These findings are inconsistent with previous studies that failed to find an association between PA and gray matter volume in the hippocampus in adolescents (Herting et al., 2016; Ruotsalainen et al., 2019). A potential explanation for this difference is that these two studies used self-reported instruments to measure participants’ PA, which differed from the objective assessment method.

Rodriguez-Ayllon et al. (2020a) also used accelerometers to objectively measure PA and SB, but they required children to wear two accelerometers, located on the right hip and nondominant wrist for 7 consecutive days (24 h/day). The total time in minutes per day consisted of LPA, MVPA, and SED. Additionally, the authors applied the Youth Activity Profile Spain (YAP-S) to evaluate the information on subjects’ self-reported PA and SB. DTI was used to capture the white matter microstructure. The authors showed that when concerning objectively measured PA, there were positive correlations of LPA, MVPA and total PA with global FA. In terms of the self-reported measures, self-reported total PA was positively correlated with global FA, whereas watching television, a self-reported SB, was negatively correlated with global FA.

All activity during waking hours can be classified into categories: SB is the opposite of PA, and these two behaviors make up all of people’s waking hours. Zavala-Crichton et al. (2020) focused on SB, brain structure and intelligence in 8 to 11-year-old overweight and obese children as part of the ActiveBrains project. SB was assessed by using the YAP-S questionnaire. Children were asked how much time, on average, they spent performing four sedentary activities (i.e., watching television, playing video games, using a cell phone, and using a computer), and total SB was calculated for each day in a week. The authors found that watching television was inversely related to gray matter volume in frontal, parietal, and occipital regions, while playing more video games was associated with reductions in gray matter volume in temporal regions. Additionally, there was a negative correlation between the total SED and gray matter volume in the cerebellum; in turn, greater gray matter volume in the cerebellar crus II was associated with a higher crystallized intelligence score. One study regarding the relationship between SB s and gray matter volume in children showed mixed results. Takeuchi et al. (2015) discovered that watching television was positively correlated with gray matter volume in frontopolar and medial prefrontal areas.

Mora-Gonzalez et al. (2019a) also used an accelerometer to objectively measure participants’ PA and sedentary time. Subjects wore two accelerometers on their nondominant wrist and right hip simultaneously for 7 consecutive days (24 h/day). The domains of cognitive flexibility and inhibition were evaluated through different tests from the nine subscales of the Delis–Kaplan Executive Function System, and planning ability was examined through the Zoo Map Test from the Behavioral Assessment of Dysexecutive Syndrome. Researchers showed that there was no significant association between PA, sedentary time and all domains of executive function indicators in overweight and obese children. These findings are contrary to the study of Davis et al. (2015) which found that compared with normal weight children who performed less PA, the more active normal weight children had higher planning scores, which indicated better executive function.

Mora-Gonzalez et al. (2020) assessed participants’ PA using accelerometers worn on the hip and nondominant wrist. The participants were 8 to 11-year-old overweight or obese children. Neuroelectric activity (i.e., P3 amplitude and latency) was registered using an electroencephalogram. The authors used a flanker task to evaluate participants’ inhibitory control, which measures a subset of cognitive function. They observed associations of PA (moderate, vigorous, and moderate to-vigorous) with inhibitory control and underlying brain activity in overweight and obese children.

Mora-Gonzalez et al. (2019b) utilized the same method to assess participants’ PA and neuroelectric activity as the study by Mora-Gonzalez et al. (2020). The authors assessed participants’ working memory using the delayed nonmatching-to-sample task. They found that vigorous PA was correlated with a higher response accuracy on the working memory task and with a larger P3 amplitude in overweight and obese children.

The above studies that observed the association of PA with brain structure and cognition in overweight and obese children were cross-sectional studies. Cross-sectional studies were inexpensive and require less time than the longitudinal studies, and their samples are usually taken from the whole population. However, it is difficult for cross-sectional studies to make causal inferences; moreover, the situation may provide different results if another time-frame is chosen (Levin, 2006).

## Exercise, cognition, and the structure and activation of the brain in obese children

### Studies of acute physical exercise interventions

The observational research mentioned above is plausibly insufficient to establish causal links. In contrast, intervention studies that employ either short or long-term physical exercise interventions, are better able to show the causal effects of exercise on the brain and cognitive abilities.

Tomporowski et al. (2008) recruited 69 children between 7 and 11 years of age who were overweight and inactive (did not take part in a regular PA program more than 1 h per week) as participants. After being randomly assigned to the exercise group and control group, participants were required to perform a 23 min treadmill walk or remained sedentary and watched an age-appropriate video for 23 min. These children performed a visual switching task in a private room before and after the intervention. However, after the exercise intervention, the researchers failed to detect the impact of an acute bout of exercise on task switching performance in the exercise group. These results are consistent with studies that failed to examine single bouts of moderately intense PA affecting task switching performance in adults (Kubesch et al., 2003).

Raine et al. (2020) also conducted a study employing a bout of acute moderate-intensity aerobic exercise in preadolescent children. A total of 116 children aged 8–11 years were recruited as participants. Participants were randomly separated into two different experimental conditions: the exercise intervention condition and the resting control condition. The exercise intervention group conducted 20 min of treadmill walking, while the resting control group was reading in a seated position silently for 20 min. After each intervention, children were instructed to perform a flanker task that required inhibitory control; reaction time and accuracy were calculated and recorded. The authors found that children showed better inhibitory control after acute moderate aerobic exercise than after the same duration of seated reading. However, these beneficial outcomes of exercise interventions seem to be attenuated in those children who have a higher BMI, which suggests that if children are extremely obese, they will not fully obtain the acute benefits of aerobic exercise on cognitive functioning.

### Studies of long-term physical exercise interventions

Many studies have also discovered a long-term exercise intervention effect on brain structure, brain activation, and cognitive function in overweight children. Davis et al. (2007) selected overweight children aged 7–11 years who attended no regular PA program more than 1 h per week as participants, and they were randomly assigned to a low-dose (20 min/day) or high-dose (40 min/day) aerobic exercise group, or a no exercise control group. The low-dose and high-dose exercise groups engaged in activities including running games, jump rope, and modified basketball and soccer, while the sedentary control group was not offered any after-school program. The children in the exercise group attended this intervention program for 5 days per week for 15 weeks. Participants’ cognitive performance was evaluated before and after the exercise program using the Cognitive Assessment System (CAS), which consists of four scales measuring different classes of cognitive processes: planning, attention, simultaneous, and successive processes. Of these scales, only the planning scale reflects executive function. The authors found that the control group had a significantly decreased posttest score relative to the high-dose exercise group; these group differences appeared only on the CAS planning scale and not on the attention, simultaneous, or successive scale. However, there were no significant posttest score differences in planning between the low-dose group and high-dose group or the control group and low-dose group. These findings suggested that children in the high-dose exercise group had greater improvements in executive function than children in the nonexercise control condition.

Long-term exercise alters the brain activation of overweight children as they perform cognitive tasks, and it also improves their academic performance. Davis et al. (2011) utilized the same aerobic exercise intervention program and recruited identical participants as the study of Davis et al. (2007). This study used fMRI to detect participants’ brain activation while completing an antisaccade task that required executive function. There were fMRI brain scans before and after the exercise intervention in the control group and the exercise group, and participants were randomly separated into low- and high-dose exercise groups. Participants’ academic achievement was examined by administrating the Woodcock–Johnson Tests of Achievement III, which contained the Broad Reading and Broad Mathematics tests. The researchers found increased bilateral prefrontal cortex activity and decreased bilateral posterior parietal cortex activity in the exercise groups compared with the control group from baseline to posttest. Moreover, a dose-dependent benefit of exercise on mathematics achievement was observed, but there was no significant difference in mathematics achievement between the exercise groups and the control group. The findings of this study are consistent with those that investigated exercise stimulated alterations in brain activity and behavioral changes in adults (Colcombe et al., 2004; Pereira et al., 2007).

Krafft et al. (2014c) recruited 43 overweight children aged 8–11 years, who were randomly divided into an aerobic exercise condition or an attention control condition. Both groups were provided an instructor-led after-school program every school day, 40 min per day for 8 months. The aerobic exercise group took part in instructor-led aerobic activities, for example, tag and jump rope, while the attention control group participated in instructor-led sedentary activities, such as art and board games. Body fat was measured with a dual-energy X-ray absorptiometry (DXA) scan. No group-by-time interactions were observed for adiposity in the participants. Before and after the intervention, fMRI data were collected while participants were performing two cognitive control tasks: the antisaccade and flanker tasks. The investigators found that relative to the sedentary control group, the exercise group showed increased activation over time in brain regions contributing to the flanker tasks, including the anterior cingulate and superior frontal gyri, and decreased activation over time in regions contributing to the antisaccade tasks, including the precentral gyrus and posterior parietal cortex. In summary, this study presents novel causal evidence that regular aerobic exercise programs alter brain function on two different cognitive tasks in overweight children.

Long-term exercise enhances white matter integrity in overweight children. Schaeffer et al. (2014) utilized the same exercise intervention program and selected a subset of participants from the study of Krafft et al. (2014c) mentioned above, but this study concentrated on white matter microstructure. At baseline, the exercise group and attention control group did not significantly differ in BMI or percent body fat. From baseline to posttest, the exercise group showed significantly decreased body fat compared with the control group. However, the BMI did not differ significantly between the two groups at the posttest assessment. The authors found that participants in the exercise group had significantly higher FA values in the bilateral uncinate fasciculus and lower radial diffusivity (RD) values in the left uncinate fasciculus, a frontotemporal white matter fiber tract, than the sedentary control group when normalized to baseline values. These findings are in line with previous research demonstrating a positive influence of long-term aerobic exercise intervention on white matter integrity in adults (Voss et al., 2013).

Krafft et al. (2014b) applied the same aerobic exercise program and followed the same participants as the study of Schaeffer et al. (2014). However, these researchers concentrated on the association between participants’ attendance of the intervention and frontoparietal white matter integrity in overweight children. The authors demonstrated that in an 8-month exercise intervention in overweight children, a group × time × attendance interaction was found. However, such an interaction was noted only in the exercise group and not in the control group. Specifically, in the exercise group, greater attendance was correlated with increased white matter integrity, namely, increased FA and decreased RD, in the bilateral superior longitudinal fasciculus (SLF) compared with the control group.

Exercise also alters the resting-state synchrony in overweight children. Krafft et al. (2014a) focused on the exercise intervention effect on resting-state synchrony in overweight children. The investigators also performed the same 8-month after-school exercise program for 22 overweight children aged 8 to 11-years who were randomly divided into an exercise group and a sedentary control group. Before and after the 8-month programs, all participants underwent resting-state fMRI scans. Resting-state fMRI analyses were performed on four networks, namely, the default-mode, salience, cognitive control, and motor networks. The authors found that the exercise group showed decreased synchrony over time in the default-mode network, cognitive control network, and motor network but increased synchrony between the motor network and a frontal region compared with the control group.

To the best of our knowledge, no study has observed that physical exercise changes the gray matter volume, cortical thickness, or surface area in overweight and obese children. However, there was evidence showing changes in gray and white matter volume in older adults after physical exercise (Colcombe et al., 2006; Matura et al., 2017).

Regarding the pathogenesis of physical exercise, studies using rodent models have shown that exercise alleviates obesity-induced metabolic disorders by affecting the expression of fibroblast growth factor 21 (FGF21) (Geng et al., 2019).

Park et al. (2019) used fed male mice a high-fat diet for 20 weeks, causing the mice to become obese. Then, the high-fat diet mice performed a treadmill exercise for 12 weeks. The authors observed that the high-fat diet mice showed a decrease in insulin signaling and neuroplasticity in the hippocampus and the dentate gyrus and impaired cognitive function, which were reversed by physical exercise.

## Discussion

In recent years, as neuroimaging technologies have developed, researchers are more likely to be able to further explore and understand the brains of children, and the research selected in this literature review mainly used MRI and fMRI to observe differences in brain structure and activation in obese children.

Altogether, we summarized the existing research and obtained mixed results of alterations in the brain, including gray and white matter structures and cortical thickness, in overweight and obese children compared with their normal-weight peers for the first time. The possible reason for these mixed results across studies may be due to the way obesity was identified and measured. An individual’s body mass is composed of both fat and lean mass; consequently, BMI does not directly measure body fat (van den Berg et al., 2013; Wright et al., 2022). The majority of previous research used BMI as the indicator to classify whether the participants were normal weight, overweight, or obese and assessed whether BMI was correlated with an individual’s brain structure and cognitive functioning (Saute et al., 2018; Laurent et al., 2020; Ronan et al., 2020). Therefore, it is difficult to tell whether previous associations were due to the fat or lean components of the body. In the study by Gracia-Marco et al. (2020), the researchers showed that there was a positive association between LMI and gray matter volume in several brain regions in overweight children, but this association was not found when BMI was used. This may suggest that BMI alone does not sensitively reflect body composition in overweight and obese children (Ortega et al., 2016b; Hinton et al., 2017); thus, using BMI may not be sensitive enough to explore all obesity-related abnormalities in the brain. To the best of our knowledge, few studies have concentrated on the relationship between LMI and brain structure. Future studies can examine the associations between LMI and total and regional gray and white matter volumes in different populations, and the findings may explain some of the previously reported heterogeneity across studies.

The time that overweight children spend in daily PA and SB s is associated with their brain structure and cognitive abilities (Mora-Gonzalez et al., 2019a; Zavala-Crichton et al., 2020). Objective assessment and self-reports are two common methods of measuring PA and SB. Self-reported measures are less accurate than objective assessments; therefore, recent studies have used accelerometers to objectively assess PA and SB (Migueles et al., 2020; Mora-Gonzalez et al., 2020). Nevertheless, accelerometers merely record the time children spend in total SB; the time spent in a specific behavior (e.g., watching television, playing video games, or using a computer or cell phone) is unlikely to be measured from accelerometer data alone (Rodriguez-Ayllon et al., 2020a). Future studies may focus on developing an initiative to objectively assess the time that individuals spend in daily specific SB, replacing the existing self-report questionnaire method, which is less accurate.

Obese children show poorer cognitive functioning than their healthy weight peers, including deficits in inhibition (Kamijo et al., 2012; Reyes et al., 2015), cognitive flexibility (Wirt et al., 2015), attention (Cserjési et al., 2007), and academic achievement (Datar and Sturm, 2006). During childhood, inhibition is particularly important because it allows children to inhibit impulsive behavior, and helps children to prolong attention, which leads to better academic performance (St Clair-Thompson and Gathercole, 2006). Children with attention deficit and hyperactivity disorder (ADHD) exhibit increased inhibitory control after an acute bout of physical exercise (Ludyga et al., 2017). In children with autism spectrum disorder, benefits were observed in inhibitory control after acute physical exercise (Bremer et al., 2020). These findings suggested that the greatest improvement of acute PA appeared in children who needed it most (i.e., lower performers) (Drollette et al., 2014). However, one study investigated whether greater BMI was associated with decreased inhibitory control after acute physical exercise (Davis et al., 2007) and suggested that the effects on children with ADHD, autism, and lower performance did not expand to children with a higher BMI. Acute bout of physical exercise is beneficial to improving inhibitory control, but the improvement is weakened as children’s BMI increases. This inconsistent result should be a cause for concern because physical exercise is possibly ineffective for those children with the highest BMI. Future studies should explore whether the negative relationship between BMI and inhibitory control still exists in children following a long-term exercise intervention, and to determine whether this relationship exists for other aspects of cognition (e.g., cognitive flexibility and attention) in addition to inhibitory control.

In the study by Schaeffer et al. (2014), the researchers used MRI methods to measure the changes in the white matter integrity of overweight children after a long-term physical exercise intervention. Long-term exercise increases white matter integrity in overweight children. On the one hand, motor training may affect white matter integrity in children. For example, a MRI study found that children who practice piano showed increased FA in frontal fiber tracts compared with their peers in the control group (Bengtsson et al., 2005). On the other hand, previous studies reported an association between physical fitness and white matter microstructure in overweight and obese children (Rodriguez-Ayllon et al., 2020b). Thus, researchers have not determined whether the changes in white matter integrity in overweight children following long-term physical exercise are due to complex motor skill training or improvements in physical fitness. Future studies may explore which factors mentioned above play a more crucial role in affecting white matter integrity in obese children. Most previous studies in children selected to perform the aerobic exercise intervention (Cao et al., 2021; Lee, 2021) whereas few studies selected anaerobic exercise, such as strength training (Marson et al., 2016; García-Hermoso et al., 2018; Chen et al., 2021). However, physically healthy children over 7 or 8 years old can perform light strength training activities such as pushups and sit-ups, following safety instructions and under adults’ supervision. Weightlifting and bodybuilding, which require very heavy weights, are not recommended for children. If children perform strength training properly and safely, they may increase their muscle strength and bone mineral density (Faigenbaum and Myer, 2010; Comité Nacional, de Medicina, del Deporte, and Infantojuvenil., 2018; Stricker et al., 2020). Future research can utilize appropriate strength training interventions for overweight and obese children to determine their influence on children’s brain health and cognition.

To the best of our knowledge, most intervention studies on this topic involved scanning children’s brains and conducting cognitive tests again immediately after the exercise intervention (Tomporowski et al., 2008; Raine et al., 2020), whereas few studies performed a subsequent postintervention follow-up to explore whether the effects of exercise on brain structure and function were sustained in the long-term, or quickly returned to a baseline level (Delisle Nyström et al., 2018; Whooten et al., 2018). If the exercise benefits last for a long time, the corresponding exercise frequency required to maintain positive outcomes can be determined. However, the exact duration of the beneficial effects of physical exercise for overweight and obese children remains unknown and future studies may examine whether exercise improvements have a short-term or long-term effects in children.

Exercise is usually regarded as an outdoor activity but more interest has been expressed in maintaining an active lifestyle for overweight and obese children at home in recent years (Arun Babu, 2022). Home exercise including sit-ups, yoga, stretching, etc., is less vigorous, requires little space and no equipment, can be safely and easily performed and should be suggested for children at home. As recreational exercise is more attractive to children than strictly fitness-oriented exercise, some exercise games, such as squat relays, indoor ball games, skipping tags, and bear crawl, are also recommended (Yu et al., 2018). However, no evidence is available to confirm the most beneficial type, intensity, or duration of exercise for obese children to perform at home as a method to attenuate the changes in their brain and cognition caused by obesity. Since different studies used various exercise programs, comparing different researchers’ findings is impossible if the variables are not controlled. Future studies should compare the effects of various exercise programs and explore the most beneficial exercise programs that are suitable for obese children to perform at home.

The prevalence of childhood obesity has increased eightfold since 1975, affecting the public healthcare system, families and communities (Grant-Guimaraes et al., 2016; Kumar and Kelly, 2017; Weihrauch-Blüher and Wiegand, 2018). Childhood is a sensitive period for brain and cognitive development (Gilmore et al., 2018) and both PA and exercise are beneficial for children regardless of their weight status (Hillman et al., 2008). Exercise can help children maintain a healthy weight and even attenuate the alterations of brain and cognition caused by obesity. We suggest that children, especially those with obesity, increase their daily physical exercise, decrease SB, and develop an active lifestyle. Overall, exercise should be a regular activity for children, and establishing an enjoyable exercise routine may help in the long-term.

## Author contributions

XS, LT, and LH contributed to conception and design of the review and wrote the manuscript. All authors contributed to the article and approved the submitted version.
